# First‐in‐human assessment of PRX002, an anti–α‐synuclein monoclonal antibody, in healthy volunteers

**DOI:** 10.1002/mds.26878

**Published:** 2016-11-25

**Authors:** Dale B. Schenk, Martin Koller, Daniel K. Ness, Sue G. Griffith, Michael Grundman, Wagner Zago, Jay Soto, George Atiee, Susanne Ostrowitzki, Gene G. Kinney

**Affiliations:** ^1^ Prothena Biosciences IncSouth San FranciscoCaliforniaUSA; ^2^ ClinPharma ResourcesSan DiegoCaliforniaUSA; ^3^ Global R&D Partners, LLCSan DiegoCaliforniaUSA; ^4^ University of CaliforniaSan Diego, San DiegoCaliforniaUSA; ^5^ Worldwide Clinical Trials, IncSan AntonioTexasUSA; ^6^ F. Hoffmann‐La Roche LtdBaselSwitzerland

**Keywords:** Parkinson's disease, clinical trial, protein aggregation, protein misfolding, synucleinopathy

## Abstract

**Background**: α‐Synuclein is a major component of pathologic inclusions that characterize Parkinson's disease. PRX002 is an antibody that targets α‐synuclein, and its murine parent antibody 9E4 has been shown in preclinical studies to reduce α‐synuclein pathology and to protect against cognitive and motor deteriorations and progressive neurodegeneration in human α‐synuclein transgenic mice. **Methods**: This first‐in‐human, randomized, double‐blind, placebo‐controlled, phase 1 study assessed the impact of PRX002 administered to 40 healthy participants in 5 ascending‐dose cohorts (n = 8/cohort) in which participants were randomly assigned to receive a single intravenous infusion of study drug (0.3, 1, 3, 10, or 30 mg/kg; n = 6/cohort) or placebo (n = 2/cohort). **Results**: PRX002 demonstrated favorable safety, tolerability, and pharmacokinetic profiles at all doses tested, with no immunogenicity. No serious adverse events, discontinuations as a result of adverse events, or dose‐limiting toxicities were reported. Serum PRX002 exposure was dose proportional; the average terminal half‐life across all doses was 18.2 days. A significant dose‐dependent reduction in free serum α‐synuclein (unbound to PRX002) was apparent within 1 hour after PRX002 administration, whereas total α‐synuclein (free plus bound) increased dose‐dependently, presumably because of the expected change in kinetics following antibody binding. **Conclusions**: This study demonstrates that serum α‐synuclein can be safely modulated in a dose‐dependent manner after single intravenous infusions of an anti–α‐synuclein antibody. These findings support continued development of PRX002, including further characterization of its safety, tolerability, pharmacokinetics, and pharmacodynamic effects in the central nervous system in patients with Parkinson's disease. © 2016 The Authors. Movement Disorders published by Wiley Periodicals, Inc. on behalf of International Parkinson and Movement Disorder Society.

Parkinson's disease is a neurodegenerative disorder that manifests a spectrum of motor, psychiatric, cognitive, sleep, and autonomic signs and symptoms. The key underlying motor manifestation is caused by a slow and progressive degeneration of dopamine‐producing neurons in the substantia nigra.^1^, ^2^ Parkinson's disease affects 7 to 10 million persons worldwide,^3^ making it the second most prevalent neurodegenerative disorder after Alzheimer's disease.^4^ Parkinson's disease is also associated with significant economic burden; in 2010, in the United States, medical expenses related to Parkinson's disease were approximately 2‐fold higher than they were for an age‐matched population without Parkinson's disease.^5^

Currently available treatments for Parkinson's disease target the dopaminergic features of the disease but do little to address its nondopaminergic symptoms and fail to treat the underlying neurodegeneration and progressive decline in neurologic function. In addition, nonmotor symptoms of the disease (such as psychosis, sleep behavior disorder, gastrointestinal dysfunction, and cognitive impairment), which can be direct or indirect outcomes of dopaminergic and other neuronal loss, are often resistant to dopamine replacement strategies and may be exacerbated by treatment under some conditions.^6^, ^7^ Furthermore, with prolonged use, currently available treatments are often associated with eventual disabling fluctuations, dyskinesias, and dose‐limiting side effects that decrease their benefits.^8^, ^9^ There is a significant unmet need for disease‐modifying therapeutic approaches that potentially slow or halt the progression of Parkinson's disease, thereby reducing the substantial personal and economic burdens it creates.

The aggregation‐prone α‐synuclein protein is the major component of Lewy bodies and Lewy neurites, which are neuropathologic hallmarks of Parkinson's disease and other neurodegenerative diseases.^10^, ^11^, ^12^ Missense mutations in the α‐synuclein gene and the overexpression of the nonmutated protein because of gene duplication or triplication are associated with early onset Parkinson's disease.^13^ Furthermore, strong correlations between clinical manifestations in Parkinson's disease and the presence and severity of α‐synuclein pathology in the brain and peripheral nerves have been reported.^14^ In preclinical studies, transgenic mice that overexpress α‐synuclein with missense mutations demonstrate a number of key features of the disease.^15^, ^16^, ^17^

Although the etiology of Parkinson's disease is yet to be determined, substantial clinical and nonclinical data suggest that soluble aggregated forms of α‐synuclein (eg, oligomers, soluble protofibrils) self‐propagate and may spread between interconnected nervous system regions and contribute to disease progression. For instance, the pattern of Lewy pathology in patients with Parkinson's disease is generally consistent with disease propagation over interconnected neuronal networks^14^; embryonic mesencephalic neurons transplanted into Parkinson's disease patients develop α‐synuclein pathology a decade after initial grafting,^18^, ^19^ and intracerebral injection of aggregated α‐synuclein accelerates the onset of neurologic symptoms and death in transgenic mice expressing human α‐synuclein.^20^ In addition to the established role of Lewy bodies, soluble aggregated α‐synuclein species have also been proposed as a major neurotoxic form of the protein in the pathophysiology of Parkinson's disease.^21^ Altogether, these genetic, neuropathologic, and nonclinical data support the therapeutic potential of agents that target aggregated forms of α‐synuclein and block the cell‐to‐cell transmission of α‐synuclein in patients with Parkinson's disease.

We have developed a monoclonal immunoglobulin G1 antibody, PRX002, designed to preferentially target soluble and insoluble aggregated forms of α‐synuclein. PRX002 is derived from the murine monoclonal antibody 9E4, which was developed based on results of immunization experiments that showed that antibodies directed against carboxyl terminus epitopes of α‐synuclein were the most effective at reducing both the neuronal accumulation of α‐synuclein and the behavioral deterioration in animal models.^15^, ^16^ For these reasons, PRX002 is being pursued therapeutically in Parkinson's disease for its potential to reduce the underlying cause of the disease and its potential ability to alter overall disease progression. Here we report the results of a first‐in‐human, double‐blind, placebo‐controlled, single ascending‐dose, phase 1 study of PRX002 that evaluated the safety, tolerability, and pharmacokinetics of PRX002 in healthy participants. Secondary and exploratory objectives assessed the immunogenicity and pharmacodynamic effects of PRX002 on serum α‐synuclein.

## Methods

### Experimental Design

This was a single‐center, randomized, double‐blind, placebo‐controlled, single ascending‐dose, phase 1 study in healthy volunteers (ClinicalTrials.gov, NCT02095171). The study was performed at Worldwide Clinical Trials Early Phase Services, LLC, San Antonio, Texas. Participants were enrolled into 1 of 5 dose‐level cohorts in which they received PRX002 (0.3, 1, 3, 10, or 30 mg/kg) or placebo administered as a 60‐minute intravenous infusion. The study duration was 16 weeks, comprising a 4‐week screening period and a 12‐week follow‐up period after administration of a single dose of the study drug. The study protocol was approved by the investigational review board for the study site. All participants provided written informed consent. The study was conducted according to International Committee on Harmonisation Good Clinical Practice guidelines and the principles of the Declaration of Helsinki.

#### Participants

Key enrollment criteria included the following: 21 to 65 years of age; body weight > 45 to ≤ 110 kg and body mass index 18 to 32 kg/m^2^; good general health with no clinically relevant abnormalities based on medical history, physical examination, clinical laboratory evaluations, and 12‐lead electrocardiography; surgically sterile or using adequate contraception; not taking medications (other than allowable oral and implanted contraceptives) unless approved by the sponsor and the investigator; and no vaccination within 30 days before baseline.

#### Randomization

Within each cohort, participants were randomly assigned in 2 blocks. In the first block, 2 participants received PRX002 or placebo 1:1 and served as sentinel participants to assess safety and tolerability before the second block was randomly assigned. In the second block, 6 participants were randomly assigned to receive PRX002 or placebo 5:1 at least 7 days after the sentinel participants were dosed and their safety data were reviewed.

#### Blinding

The study participants, investigators, sponsor, and all staff members involved in the conduct of the study were blinded to treatment assignment. The only personnel who had access to the randomization code before database lock were the pharmacist and a bioanalyst/pharmacokineticist not involved in the clinical assessment of the participants. Randomization codes were maintained in a secure location to which only the nonblinded staff had access.

#### Procedures

All participants were screened within 4 weeks before administration of the study drug. Eligible participants were confined in the study unit from the day before dosing until 24 hours after dosing. Participants returned at intervals for outpatient visits until the final assessment at week 12. Dose escalation for the second cohort was based on the evaluation of 4 weeks of safety data from the first cohort; for each subsequent cohort, dose escalation was based on 2 weeks of safety data from the preceding cohort.

PRX002 is a humanized IgG1 monoclonal antibody. The manufacturing process for PRX002 drug substance used a recombinant Chinese hamster ovary cell line grown in a suspension culture. The manufacturing campaign consisted of cell culture and harvest operations followed by a multiple‐step purification and formulation process. Manufactured PRX002 had the expected structure and biological activity demonstrated by the results of an extensive and comprehensive evaluation using an array of accepted analytical techniques. All drug substances and drug products were produced under good manufacturing practice conditions. PRX002 was supplied by the sponsor in sterile vials containing 200 mg lyophilized PRX002, which was reconstituted with water for injection. Reconstituted PRX002 was mixed with normal saline to obtain a total volume of 250 mL. The study drug was prepared by a nonblinded study pharmacist for intravenous infusion over 60 ± 10 minutes. Normal saline (250 mL) was used as the placebo.

All participants were asked to return 3 days, 7 days (week 1), 14 days (week 2), 28 days (week 4), 42 days (week 6), and 84 days (week 12) after dosing for clinical, safety, and pharmacokinetic assessments. Immunogenicity was assessed before dosing and at 2, 4, and 12 weeks after dosing. Free and total serum α‐synuclein concentrations were assessed before dosing and at 1 to 3 days and at 1, 2, 4, 6, and 12 weeks after dosing. Serum PRX002 concentrations were assessed at the following times: before dosing; 30 minutes after the start of the infusion; at the end of the 60‐minute infusion; 0.25, 0.5, 1, 2, 4, and 8 hours after the end of the infusion; 1 and 3 days after dosing; and 1, 2, 4, 6, and 12 weeks after dosing. Cerebrospinal fluid was not collected in this healthy volunteer study.

#### Outcomes

The primary study objectives were to evaluate the safety, tolerability, and pharmacokinetics of a single intravenous infusion of PRX002, and the secondary objective was to assess immunogenicity. Safety was assessed in all participants who received the study drug (PRX002 or placebo); assessments included adverse events (AEs), serious AEs, severity of AEs, laboratory tests, vital signs, electrocardiography, and physical and neurologic examinations and were reviewed before dose escalation. Pharmacokinetic and pharmacodynamic sampling was performed on all participants. Serum PRX002 was measured using a validated, quantitative sandwich electrochemiluminescence (ECL) assay (lower limit of quantification 39.0 ng/mL) developed by Prothena. Serum PRX002 pharmacokinetic parameters were estimated using noncompartmental techniques (Pheonix WinNonlin version 6.3; Pharsight Corporation, St. Louis, Missouri), including maximum concentration, time to reach maximum concentration, area under the serum concentration‐time curve from time 0 hours to last quantifiable concentration calculated using linear trapezoidal summation, area under the serum concentration‐time curve from time 0 hours to infinity (AUC_inf_), observed terminal rate constant, terminal elimination half‐life, total body clearance, and volume of distribution.

Immunogenicity was assessed by measurement of serum anti‐PRX002 antibodies. A qualitative bridging ECL assay was used to screen for anti‐PRX002 antibodies in human serum, with positive samples subject to a confirmatory assay. Total serum α‐synuclein and free serum α‐synuclein were measured using a quantitative ECL assay using a commercially available kit (Meso Scale Discovery, Rockville, Maryland). Samples with high (>600 μg/mL) hemoglobin levels were excluded from α‐synuclein measurements.

### Statistical Analysis

Baseline and demographic characteristics, safety, and pharmacokinetic data were summarized using descriptive statistics. Total α‐synuclein and free serum α‐synuclein concentrations and change from baseline concentrations at each sampling time were summarized at each dose level using descriptive statistics and were assessed using a repeated‐measures analysis performed separately for free or total serum α‐synuclein. Two linear fixed‐effects models, adjusted for baseline as a covariate, were fit to results from 10 postbaseline time points. Treatment, time, and their interaction, estimated using a restricted maximum likelihood method, were included in the model. Given that the treatment‐by‐time interaction was significant for both parameters, the sequential trend test was performed at each time point for both the free and total α‐synuclein levels. All tests, including main and interaction effects, were conducted at the 2‐sided significance level of .05.

When relevant, participants who received placebo were pooled across cohorts. Participant disposition, discontinuation, and treatment‐emergent AEs were summarized using the frequencies and percentages of participants (by treatment and dose where appropriate).

## Results

### Study Population

A total of 40 healthy volunteers were randomly assigned to receive placebo or PRX002 from October 2014 to February 2015. Participants ranged in age from 21 to 58 years; the median age was 37.0 years (Table 1). Greater proportions of participants were women (62.5%) and white (57.5%). Median body weight was 75.4 kg. No patients had a notable medical history, and all were clinically well during the 4 weeks before the study; baseline laboratory values were normal. There were no significant protocol deviations.

**Table 1:** Baseline characteristics of healthy volunteers

Characteristic	Placebo, n = 10	PRX002					
		0.3 mg/kg, n = 6	1 mg/kg, n = 6	3 mg/kg, n = 6	10 mg/kg, n = 6	30 mg/kg, n = 6	All participants N = 40
Median age, years (range)	45.0 (30.0‐57.0)	35.0 (21.0‐45.0)	32.5 (26.0‐58.0)	33.5 (22.0‐53.0)	36.5 (21.0‐58.0)	37.0 (22.0‐56.0)	37.0 (21.0‐58.0)
Sex, n (%)							
Male	5 (50.0)	2 (33.3)	1 (16.7)	3 (50.0)	0 (0.0)	4 (66.7)	15 (37.5)
Female	5 (50.0)	4 (66.7)	5 (83.3)	3 (50.0)	6 (100.0)	2 (33.3)	25 (62.5)
Ethnicity, n (%)							
Hispanic or Latino	6 (60.0)	3 (50.0)	3 (50.0)	5 (83.3)	2 (33.3)	4 (66.7)	23 (57.5)
Not Hispanic or Latino	4 (40.0)	3 (50.0)	3 (50.0)	1 (16.7)	4 (66.7)	2 (33.3)	17 (42.5)
Race, n (%)							
White	6 (60.0)	1 (16.7)	5 (83.3)	4 (66.7)	4 (66.7)	3 (50.0)	23 (57.5)
Black or African American	4 (40.0)	4 (66.7)	1 (16.7)	1 (16.7)	2 (33.3)	2 (33.3)	14 (35.0)
Asian	–	–	–	–	–	–	–
American Indian or Alaska Native	–	1 (16.7)	–	1 (16.7)	–	–	2 (5.0)
Native Hawaiian or other Pacific Islander	–	–	–	–	–	1 (16.7)	1 (2.5)
Median body weight, kg (range)	70.9 (55.1‐83.3)	73.9 (55.0‐81.5)	77.3 (70.2‐90.9)	88.6 (55.0‐104.2)	70.3 (55.2‐82.8)	84.4 (50.4‐91.5)	75.4 (50.4‐104.2)
Median BMI, kg/m^2^ (range)	26.6 (19.2‐31.4)	26.5 (22.4‐30.5)	28.5 (25.0‐31.9)	30.5 (21.9‐31.3)	26.4 (22.7‐30.2)	27.4 (20.4‐31.2)	27.2 (19.2‐31.9)

BMI, body mass index.

### Safety

Single intravenous infusions of PRX002 ranging from 0.3 to 30 mg/kg were well tolerated, and no safety issues were identified; no deaths, serious AEs, or dose‐limiting toxicities were reported. All participants completed the study, with the exception of 1 participant randomly assigned to 0.3 mg/kg PRX002 and lost to follow‐up after the week 2 visit. Of the participants, 18 reported treatment‐emergent AEs (Table 2), most of which were mild, and 6 AEs of moderate severity were considered unrelated to the study drug (2 were in placebo recipients). Only 1 severe AE, neutropenia, was reported and was assessed by the investigator to be of viral etiology and unrelated to the study drug (10 mg/kg PRX002). The most common treatment‐emergent AEs were headache, nausea, vessel puncture site pain, viral infection, and viral upper respiratory tract infection. Salivary hypersecretion, a sense of heaviness in the left arm, headache, nausea, fatigue, and abdominal pain were the only AEs considered by the investigator to be related to study treatment (defined as any AE with an onset on or after the first dose of the study drug or any pre‐existing condition that worsened on or after the first dose of the study drug); these were mild and resolved by the end of the study. No clinically relevant or treatment‐relevant trends emerged from clinical laboratory data, physical examination, vital signs, or electrocardiography after dosing. No anti‐PRX002 antibodies were detected.

**Table 2:** Treatment‐emergent adverse events occurring in ≥5% of participants

	Placebo, n = 10	PRX002					All PRX002 doses, n = 30
		0.3 mg/kg, n = 6	1 mg/kg, n = 6	3 mg/kg, n = 6	10 mg/kg, n = 6	30 mg/kg, n = 6	
Adverse events, n (%)	5 (50.0)	1 (16.7)	1 (16.7)	3 (50.0)	4 (66.7)	4 (66.7)	13 (43.3)
Treatment‐related adverse events, n (%)	1 (10.0)	–	1 (16.7)	–	3 (50.0)	–	4 (13.3)
Treatment‐emergent adverse events, n (%)							
Vessel puncture site pain	1 (10.0)	–	–	–	1 (16.7)	1 (16.7)	2 (6.7)
Headache	1 (10.0)^a^	–	–	1 (16.7)	1 (16.7)^a^	–	2 (6.7)
Viral infection	1 (10.0)	1 (16.7)	–	–	1 (16.7)	–	2 (6.7)
Nausea	–	–	–	–	2 (33.3)^a^	–	2 (6.7)
Neutropenia	–	1 (16.7)	–	–	1 (16.7)^b^	–	2 (6.7)
Upper respiratory infection	–	–	–	–	1 (16.7)	1 (16.7)	2 (6.7)
Pruritus	1 (10.0)	–	–	–	1 (16.7)	–	1 (3.3)

Unless indicated, all adverse events were mild and unrelated to study drug.

a Considered related to study drug.

b Severe adverse event of neutropenia was reported and was determined by the investigator to be viral in origin and unrelated to study drug.

### Pharmacokinetics

**Figure 1:**
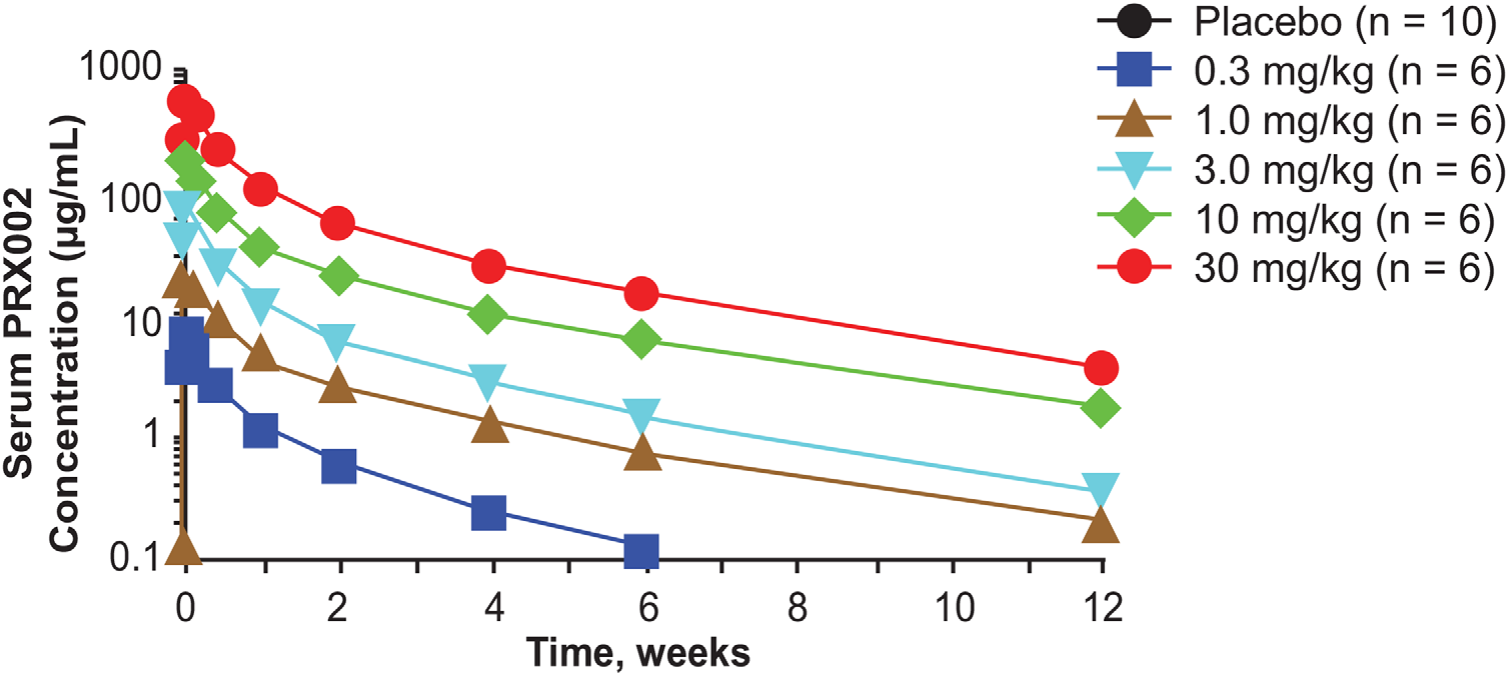
Pharmacokinetics of PRX002. Serum PRX002 concentration–time profiles after a single dose of PRX002.

Serum PRX002 exposure generally increased in parallel with PRX002 doses over the range of 0.3 to 30 mg/kg (Fig. 1; Table 3). Mean maximum concentrations ranged from 7.6 μg/mL (0.3 mg/kg PRX002) to 578 μg/mL (30 mg/kg PRX002), and mean AUC_inf_ ranged from 881 h·μg/mL (0.3 mg/kg PRX002) to 85,920 h·μg/mL (30 mg/kg PRX002). Average terminal half‐life across all doses was 18.2 days. Total body clearance was similar across doses, ranging from 0.2553 mL/h/kg (1 mg/kg PRX002) to 0.3492 mL/h/kg (30 mg/kg PRX002). Volume of distribution values ranged from 124.1 mL/kg (0.3 mg/kg) to 240.6 mL/kg (10 mg/kg PRX002).

**Table 3:** Pharmacokinetic parameters after the infusion of PRX002

Pharmacokinetic parameter	PRX002				
	0.3 mg/kg, n = 6	1 mg/kg, n = 6	3 mg/kg, n = 6	10 mg/kg, n = 6	30 mg/kg, n = 6
Measure, geometric mean (CV %)					
C_max_, µg/mL	7.6 (7.8)	22.4 (15.9)	76.4 (12.9)	194 (13.9)	578 (20.4)
AUC_last_, h·µg/mL	818 (11.2)	3774 (18.8)	9341 (23.8)	29730 (12.7)	83380 (19.1)
AUC_inf_, h·µg/mL	881 (10.8)	3918 (20.3)	9564 (23.7)	31070 (14.2)	85920 (20.6)
λ_z_, 1/h	0.0027 (51.7)	0.0014 (16.8)	0.0016 (3.6)	0.0013 (15.8)	0.0015 (20.1)
Terminal t_½_, h	252.5 (51.7)	482.0 (16.8)	441.0 (3.6)	518.1 (15.8)	459.8 (20.1)
CL, mL/h/kg	0.3406 (10.8)	0.2553 (20.25)	0.3137 (23.7)	0.3219 (14.2)	0.3492 (20.6)
V_d_, mL/kg	124.1 (55.3)	177.5 (14.7)	199.6 (23.8)	240.6 (13.6)	231.6 (14.8)
T_max_, h^a^	1.25 (1.00, 3.00)	1.17 (1.00, 2.12)	1.13 (1.00, 3.00)	2.51 (1.08, 9.02)	1.50 (1.00, 3.00)

CL, total body clearance; C_max_, maximum concentration; CV, coefficient of variation; h, hour; λ_z_, observed terminal rate constant; t_½_, terminal elimination half‐life; T_max_, time to reach C_max_; V_d_, volume of distribution.

### Pharmacodynamics

**Figure 2:**
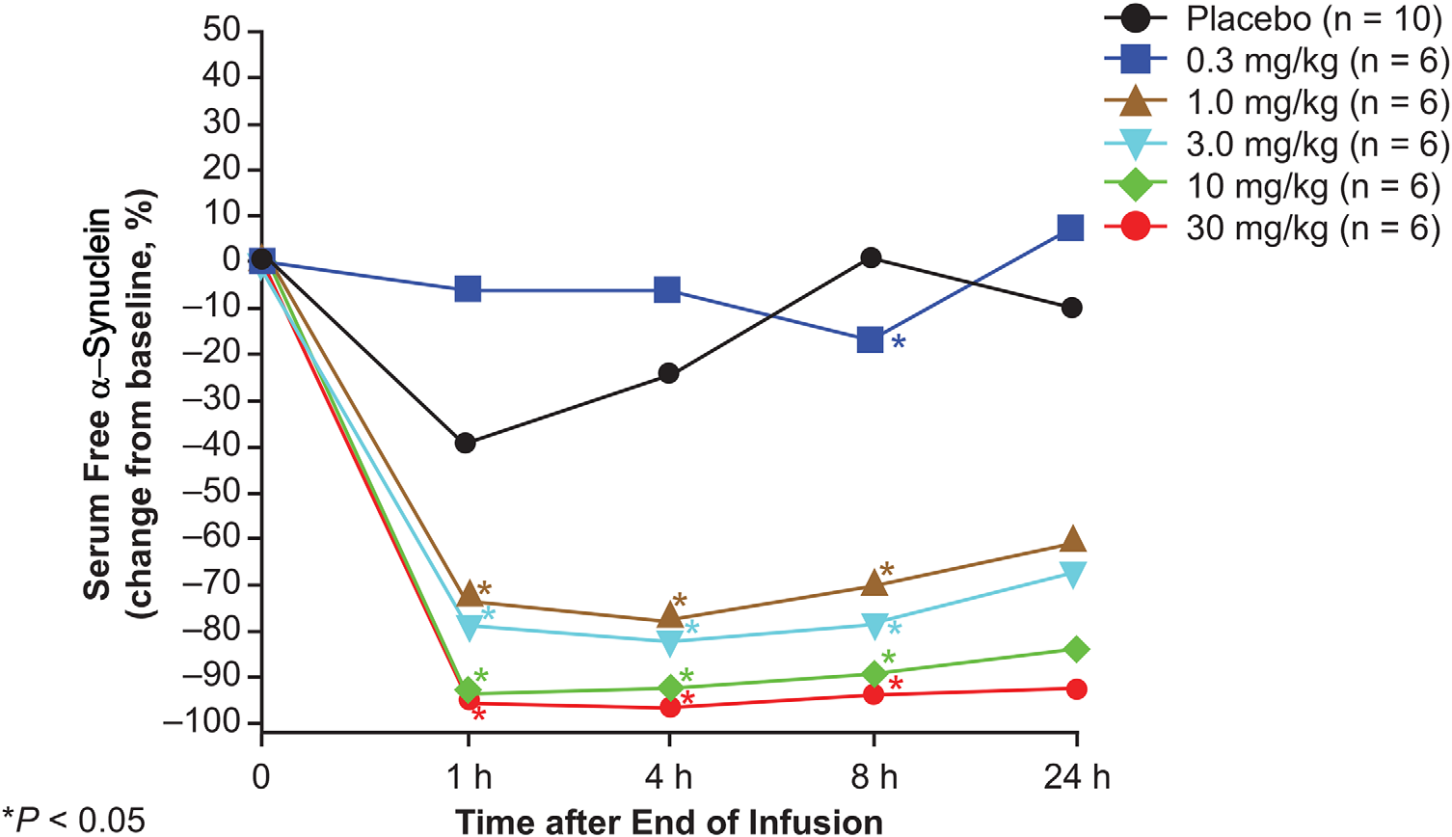
Pharmacodynamics of PRX002. Change from baseline of serum‐free α‐synuclein after a single dose of PRX002.

A dose‐ and time‐dependent, statistically significant reduction in free (PRX002 unbound) serum α‐synuclein of up to 96.5% was apparent within 1 hour after the end of the infusion, which was the first time point sampled after infusion (*P* < .0001; Fig. 2). These reductions varied in duration between doses and lasted from 2 weeks (10 mg/kg) to 4 weeks (30 mg/kg). The greatest median reduction and percentage change from baseline in serum free α‐synuclein levels at each dose were as follows: 0.3 mg/kg PRX002, −2,923 pg/mL (−17%) at 3 days; 1 mg/kg PRX002, −18,010 pg/mL (−77.6%) at 4 hours; 3 mg/kg PRX002, −12,574 pg/mL (−82.4%) at 4 hours; 10 mg/kg PRX002, −13,301 pg/mL (−93.4%) at 1 hour; and 30 mg/kg PRX002, −15,774 pg/mL (−96.5%) at 4 hours. Pharmacodynamic responses of increased total (consisting of PRX002 bound and PRX002 unbound) serum α‐synuclein were also statistically significant and were both dose and time dependent for 3‐, 10‐, and 30‐mg/kg PRX002 cohorts beginning at 1 day after dosing. The highest median percentage increases from baseline in total α‐synuclein were as follows: 3 mg/kg PRX002, 12,077 pg/mL (100.5%) at 7 days; 10 mg/kg PRX002, 20,446 pg/mL (129.2%) at 3 days; and 30 mg/kg PRX002, 27,110 pg/mL (196.9%) at week 2.

## Discussion

In this first‐in‐human study, single intravenous doses of PRX002 were safe and well tolerated and demonstrated expected pharmacokinetic profiles at all dose levels tested, up to and including 30 mg/kg. No deaths, serious AEs, dose‐limiting toxicities, or generation of anti‐PRX002 antibodies resulted from treatment. PRX002 significantly and substantially reduced free serum α‐synuclein levels within 1 hour after the end of the infusion, the first time point assessed. These reductions were persistent for 2 to 4 weeks after a single infusion. These results demonstrate for the first time that serum α‐synuclein can be safely modulated in humans in a dose‐dependent manner after single intravenous infusions of the anti–α‐synuclein antibody PRX002.

Current pharmacologic treatments for Parkinson's disease aim to attenuate the dopamine‐related symptoms of the disease, acting primarily to increase dopamine production or stability or directly stimulate postsynaptic dopamine receptors. However, in the long term, patients are served inadequately because of treatment‐related dyskinesia and the development or persistence of nondopaminergic or nonmotor symptoms such as postural instability with related falls or cognitive deficits.^6^, ^7^, ^22^, ^23^ Given the central role of α‐synuclein in the neuropathology of Parkinson's disease, a reduction in α‐synuclein production or the elimination of α‐synuclein aggregates may benefit patients with this disease.^24^

The predominant intraneuronal localization of α‐synuclein bound to presynaptic terminal membranes^25^ has supported a normal function of the protein in presynaptic vesicle supply and release and synaptic plasticity in neurons. The functions, if any, of extracellular α‐synuclein, however, are not known. Although various groups^26^, ^27^, ^28^ have described α‐synuclein levels in cerebrospinal fluid as reduced in patients with Parkinson's disease, no consistent diagnostic or prognostic connection has been made between absolute α‐synuclein levels and Parkinson's disease symptoms and/or stage in either cerebrospinal fluid or serum. Nonetheless, considering the presence of this protein in serum, it is notable that substantial reductions in free serum α‐synuclein by PRX002 were without apparent adverse physiological effect in the single‐dose setting.

Given that the present study did not include cerebrospinal fluid collections, it remains to be demonstrated in future clinical studies whether PRX002 penetrates the central compartment, as is predicted by preclinical studies. The ability of peripherally administered antibodies to cross the blood–brain barrier to act directly in the central nervous system has been shown.^29^, ^30^ Results from our previous studies in 2 transgenic mouse models of α‐synucleinopathies demonstrated that neuronal aggregation of α‐synuclein and associated loss of neuronal synapses, gliosis, and motor and cognitive behavior deficits can be reduced by treatment with the 9E4 antibody, suggesting that the antibody does access the brain in mice.^15^, ^17^ Furthermore, in patients with Alzheimer's disease, peripherally administered antibodies against β‐amyloid were shown to clear aggregated forms of that protein in the brain and to reduce a downstream marker of neurodegeneration.^28^, ^31^, ^32^ In an ongoing multiple ascending‐dose, phase 1 study of PRX002 in patients with Parkinson's disease, cerebrospinal fluid samples will be collected to determine the level of peripherally administered PRX002 in the central compartment.

Although the results of this first‐in‐human study demonstrated that PRX002 safely modulates α‐synuclein in the periphery, it is not possible at this time to conclude whether soluble monomers or aggregates are preferentially targeted in this measure. At a mechanistic level, 9E4 binds α‐synuclein aggregates with substantially higher affinity than α‐synuclein monomers^33^ to block cell‐to‐cell transmission of α‐synuclein and to limit cleavage of the protein by calpain,^15^ which generates highly amyloidogenic peptide fragments of synuclein in Lewy bodies.^34^ In addition, 9E4 demonstrated superior efficacy compared with other antibodies that target a number of other epitopes in α‐synuclein. Improved efficacy may result from the antibody's high affinity for aggregated α‐synuclein and from interaction with an epitope on cytotoxic aggregates that is required for their uptake by neurons (eg, blocked binding to plasma membrane) and/or posttranslational modifications (eg, protein truncations).^15^, ^16^, ^35^ Once the antibody is bound to α‐synuclein, the complex may be cleared from the extracellular space by microglia, neuronal autophagy, or bulk flow of interstitial fluid from the brain parenchyma, which would limit further oligomerization and reduce seeding activity, cell‐to‐cell transmission, or both.^36^, ^37^ Although aggregated α‐synuclein is thought to be the relevant target of PRX002, multiple forms may exist; hence, technical challenges limit assay development and interpretation of aggregated α‐synuclein in animal and human tissue. Among several factors impeding the establishment of a reliable assay are the presumed low abundance of α‐synuclein aggregates in fluid and the fact that their primary presence occurs within the brain at sites not accessible to detection. Nevertheless, pharmacodynamic changes of the overall α‐synuclein pool caused by PRX002 can be assessed by measuring changes in serum α‐synuclein over time.

Ongoing and future clinical studies with PRX002 will aim to determine the safety and efficacy of multiple doses of PRX002 in patients with Parkinson's disease. Because the enrolled participants of the present single‐dose, healthy volunteer study were generally younger (median age, 37 years; range, 21‐58 years) and a higher proportion of them were not white (42.5%) compared with a more typical patient population with Parkinson's disease, safety and tolerability will continue to be closely assessed in future studies. Moreover, although no anti‐PRX002 antibodies were detected in this study, it remains important to continue to assess this end point in ongoing and future studies in which patients will receive multiple doses of the study drug.

This phase 1 clinical trial in healthy volunteers demonstrates the safety of a single intravenous dose of PRX002 and its ability to significantly reduce the levels of free α‐synuclein in serum. These results support the design of the ongoing, multiple‐ascending dose, double‐blind, phase 1 study evaluating PRX002 in patients with Parkinson's disease (ClinicalTrials.gov, NCT02157714).

## Author Roles

1. Research Project: A. Conception, B. Organization, C. Execution; 2. Statistical Analysis: A. Design, B. Execution, C. Review and Critique; 3. Manuscript Preparation: A. Writing the First Draft, B. Review and Critique.

D.B.S.: 1A, 2C, 3A, 3B

M.K.: 1A, 2C, 3A, 3B

D.K.N.: 1A, 2C, 3A, 3B

S.G.G.: 1A, 2C, 3A, 3B

M.G.: 1A, 2C, 3A, 3B

W.Z.: 1A, 2C, 3A, 3B

J.S.: 2C, 2C, 3A, 3B

G.A.: 2B, 3A, 3B

S.O.: 1A, 2C, 3A, 3B

G.G.K.: 1A, 2C, 3A, 3B

## Full financial disclosure for the previous 12 months

D.B.S. reported employment, leadership, stock ownership, intellectual property, and travel for Prothena. M.K. reports employment, leadership, stock ownership, and travel for Prothena. D.K.N. reports employment, stock ownership, and travel for Prothena. S.G.G. is a consultant for Prothena. M.G. is a consultant and reports stock ownership in Prothena. W.Z. reports employment, stock ownership, and travel for Prothena. J.S. reports employment, stock ownership, and travel for Prothena. G.A. has nothing to disclose. S.O. reports employee status and stock ownership in F. Hoffmann‐La Roche Ltd. G.G.K. reports employment, leadership, stock ownership, travel, and patents/intellectual property with Prothena.
